# Comparison of anxiety and depression levels in caregivers of patients with percutaneous endoscopic gastrostomy for home enteral tube feeding against other enteral nutrition methods

**DOI:** 10.1186/s12904-024-01360-3

**Published:** 2024-01-22

**Authors:** Gökmen Özceylan, İnahet Findik

**Affiliations:** Tekirdağ DRIFC State Hospital palliative service, TekİrdaĞ, Turkey

**Keywords:** Home enteral tube feeding, Percutaneous endoscopic gastrostomy, Caregiver, Hospital anxiety and depression scale

## Abstract

**Background:**

The aim of the study was to demonstrate whether the care burden of caregivers of bedridden patients, who experience feeding difficulties, decreases according to the Hospital Anxiety and Depression Scale (HADS) (1) after the patient has undergone PEG (Percutaneous Endoscopic Gastrostomy). The hypothesis of the study was that the levels of anxiety and depression of caregivers for patients fed via PEG decrease more than those for caregivers of patients fed through other enteral methods. Based on this, the goal is to recommend to palliative care and home care professionals the type of feeding method for bedridden patients that will create less burden on their relatives.

**Methods:**

A comparison was made of the Hospital Anxiety and Depression Scale (HADS) scores among caregivers of patients receiving PEG and other enteral nutrition, focusing on changes between hospital admission and discharge. These changes were analyzed based on the caregiver’s age, gender, and the duration of the patient’s hospital stay.

**Methods:**

This study conducted a comparative analysis of the Hospital Anxiety and Depression Scale (HADS) scores among caregivers of patients receiving Percutaneous Endoscopic Gastrostomy (PEG) versus other forms of enteral nutrition. The focus was on the variation in these scores from the time of the patients’ hospital admission to their discharge. This analysis incorporated an examination of how these changes correlated with the caregiver’s age and gender, as well as the duration of the patient’s hospitalization.

**Results:**

Despite longer hospital stays, a decrease in anxiety and depression was observed in caregivers of patients receiving PEG compared to the other group (*p* = 0.078). It was found that the decrease in anxiety and depression levels was less pronounced with increasing age of the caregiver (*r*=-0.202, *p* = 0.038). Hospital stay duration for patients receiving PEG was significantly longer than for those receiving other enteral nutrition methods (*p* = 0.017).

**Conclusions:**

We believe that Percutaneous Endoscopic Gastrostomy (PEG) should be the preferred method for long-term enteral nutrition due to its facilitation of effective and comfortable feeding and medication administration by caregivers. In palliative care services, for patients requiring long-term nutrition, PEG should be considered more prominently than other enteral feeding methods to reduce the anxiety of caregivers.

## Introduction

The aging population is increasing worldwide, and advancements in medical technologies are leading to longer life spans and the emergence of complex health problems [1]. Difficulty in self-feeding naturally is a major issue among elderly patients. In recent years, Home Enteral Tube Feeding (HETF) has emerged as a significant solution to this problem, with an increasing number of patients being managed [2]. The rapid development of technology and the proliferation of home health services have transformed enteral tube feeding from a hospital-based method to one that allows patients to continue living in their home environment with their families. HETF is increasingly preferred by health professionals and patients relatives for reducing hospital infections, readmissions, unnecessary occupation of hospital beds, and costs [3]. Enteral nutrition, when feasible, presents numerous advantages over parenteral nutrition due to its closer resemblance to natural feeding. These advantages include nourishing and supporting the immune system, as well as promoting its protection and maintenance. It also benefits the gastrointestinal system. Additionally, enteral nutrition is more cost-effective and easier to administer [4–6].

The selection of an enteral nutrition method for a patient is contingent upon the duration for which enteral feeding is required and the length of time the patient will need care. This decision-making process should consider both the nutritional needs and the expected period of dependency on enteral feeding If the planned duration is less than four weeks, methods like nasogastric (NG), nasoduodenal, nasojejunal, or orogastric tube feeding are selected. For durations longer than four weeks, methods like Percutaneous Endoscopic Gastrostomy (PEG), Percutaneous Endoscopic Jejunostomy (PEJ), surgical gastrostomy, or surgical jejunostomy are chosen [7].

Study aims to measure and compare the anxiety and depression status of caregivers of patients nourished through PEG and other enteral nutrition methods.

## Methods

The study was approved by Tekirdağ DrİFC City Hospital’s Non-Interventional Ethics Committee, with approval obtained on 31.01.2023, No. 24. It involved a retrospective examination of hospital data from January to December 2022. The study was conducted using data from the Hospital Anxiety and Depression Scale (HADS), a routine assessment administered to both patients and their caregivers upon admission and at discharge in the palliative care unit. This approach facilitated an in-depth analysis of the psychological impact of palliative care on both patients and caregivers during their stay in the unit. Inclusion criteria were: (For Patients) - Inability to feed orally, - Requirement for enteral feeding. (For Caregivers) - Caring for the patient for at least one week or longer, - Continuous caregiving throughout the patient’s stay in the palliative unit, - No history of psychiatric illness.

In the palliative unit, the HADS is routinely applied to caregivers within the first 24 h of a patient’s admission and again at discharge. The study retrospectively examined the scale data of caregivers who met the inclusion criteria.

HADS, utilized in palliative care unit, is a self-assessment tool designed to identify and measure the level and severity of anxiety and depression among patients and caregivers in the hospital setting (Zigmond & Snaith, 1983). It comprises a total of 14 questions, with seven assessing anxiety and the remaining seven measuring depression. Following the validity and reliability study conducted by Aydemir et al. (1997), the reliability coefficient (Cronbach’s Alpha) was found to be 0.75 [8]. In this study, the HADS was employed with a scoring range from a minimum of 4 to a maximum of 1, where a higher score indicates lower levels of anxiety and depression for caregivers. This arrangement is due to its role as one of the nine sub-scales used in the assessment tool applied at admission and discharge in the palliative service where the study was conducted. These scales were initially designed to progress from the lowest to the highest entry score, eventually transforming into a tool that interprets higher scores as indicative of improved patient and caregiver conditions [9]. The study demonstrated that an increase in caregivers’ HADS scores between initial and final assessments signifies a reduction in anxiety and depression levels, while a decrease in scores indicates an increase in these levels. These differences were further analyzed based on the caregivers’ age, gender, duration of hospital stay and HADS scores.

Data obtained in the study were uploaded to the IBM Statistical SPSS 22.0 program. Besides descriptive statistical methods (mean, standard deviation, frequency), the Pearson chi-square test was employed for comparing data by caregivers’ gender. Independent t-tests and Pearson correlation tests were utilized to analyze age, duration of hospital stay, and HADS scores. Results were evaluated at a 95% confidence interval and a significance level of p < 0.05.”

## Results

A total of 105 caregivers participated in the study, with 77 of them (73.3%) being female. The average age of the participants was 54.9 ± 11.6 (min 43, max 68). The average hospital stay duration for patients and their caregivers was 20.3 ± 7.3 days (min 17, max 27).

The hospital stay duration for patients receiving PEG was significantly longer than for those receiving other enteral nutrition methods (*p* = 0.017). In the study, there was no significant difference between caregivers of patients receiving PEG and those receiving other enteral nutrition methods in terms of gender, age, initial HADS average score (IHAS), and discharge HADS average score (DHAS) (Table 1).

**Table 1:** Comparison of the gender and age distribution of caregivers, hospital stay durations, and Anxiety-Depression levels based on PEG status

Category	PEG Yes (*n* = 36)	PEG No (*n* = 69)	p-value
**Gender, n (%)**			
Male	8 (22.2%)	20 (29.0%)	*0.457*
Female	28 (77.8%)	49 (71.0%)	
Caregiver’s Age	56.13 ± 12.12	54.28 ± 11.59	*0.447*
Length of Stay	25.63 ± 13.17	19.63 ± 8.85	***0.017***
IHAS	31.47 ± 8.72	30.59 ± 8.14	*0.610*
DHAS	36.72 ± 9.54	33.57 ± 9.51	*0.112*

IHAS, Initial Hospital Anxiety and Depression Scale Score; DHAS, Discharge Hospital Anxiety and Depression Scale Score

*p* < 0.05: Statistically significant

^a^Pearson Chi-Square test n (%)

^b^Independent t-test (Mean ± SD)

In the study, Despite longer hospital stays for patients and their caregivers in the PEG group compared to the other group, a reduction in anxiety and depression among caregivers was observed (*p* = 0.078) (Table 2).

**Table 2:** Comparison of the change in average HADS scores of caregivers during the hospital stay based on PEG status

PEG Status	Yes (*n* = 36)	No (*n* = 69)	p-value
IHAS	31.47 ± 8.72	30.59 ± 8.14	0.610
DHAS	36.72 ± 9.54	33.57 ± 9.51	0.112
DHAS-IHAS	5.25 ± 5.22	2.98 ± 7.68	*0.078*

IHAS, Initial Hospital Anxiety and Depression Scale Score; DHAS, Discharge Hospital Anxiety and Depression Scale Score

In the study, a significant negative correlation was found between the change in average HADS scores of caregivers during the hospital stay and the age of the patient they cared for. It was observed that as the age of the caregiver increased, the reduction in anxiety and depression levels was less pronounced (*r*=-0.202, *p* = 0.038). (Table 3)

**Table 3:** Relationship between the change in average HADS scores of caregivers during the hospital stay and the duration of stay and age of the caregiver

		Difference	Length of stay	Caregiver’s age
**Difference**	r	1		
	p			
**Length of stay**	r	0,101	1	
	*p*	*0,305*		
**Caregiver’s age**	r	-0,202^*^	-*0,008*	1
	*p*	***0,038***	0,933	

Pearson correlation test

## Discussion

PEG was first described and implemented by Gauderer and Ponsky [10]. It has replaced surgical gastrostomy, which carries a higher risk of complications. The PEG tube is inserted with the aid of endoscopy under local anesthesia. This procedure can be performed bedside with minimal sedation, outside of the endoscopy unit; it is less complicated, requires a shorter hospital stay, and is more economical compared to surgery [11]. According to guidelines, if there is no improvement in oral intake within the first seven days, enteral feeding with an NG tube is recommended. However, if swallowing disorders are expected to last more than 30 days, PEG is preferred. Because it is more difficult for patients to unintentionally remove or dislodge a PEG compared to an NG tube. For long-term care, PEGs are often preferred over NG tubes [12]. Consistent with the literature, in this study, approximately 40% of patients in the palliative unit with feeding difficulties were chosen for enteral tube feeding (ETF) through PEG based on appropriate indications. These decisions were made considering the patients’ duration of enteral nutrition needs, comorbid conditions, and consent from the patient and their relatives.

In this study, insertion of a PEG for elderly and dependent patients unable to feed orally or achieve adequate nutrition, extended the hospital stay. Prolonged hospitalization can increase anxiety and depression in both patients and caregivers [13]. Despite this longer duration, compared to those fed through other enteral methods, all caregivers, regardless of age and gender, showed reduced hospital anxiety and depression levels with PEG. The reduction in anxiety and depression levels was less pronounced with increasing age of the caregiver. Caregivers in palliative units already face significant challenges, and the initiation of feeding through PEG, compared to other enteral methods, lessens these difficulties. We believe that as people age, their likelihood of hospitalization increases, and this leads to less variation in hospital anxiety and depression levels compared to younger individuals. We think such a result emerges from this trend.

Approximately 10% of stroke patients in Turkey whose nutritional intake was managed via PEG, representing aprproximately 15,000 patients annually, highlighting the critical importance of developing a global nutritional strategy in acute stroke [14]. In the study, the proportion of patients fed through PEG was around 40%, indicating an increasing preference for PEG in Turkey over the years. The study also found longer hospital stays for patients fed through PEG compared to those fed through other enteral methods. We believe the reason for the longer stays in the palliative service of this study, which is not consistent with the literature, is due to the prolonged anesthesia time prior to PEG and the waiting time due to only one endoscopy unit available for PEG procedures. The authors consider; In the context of hospital palliative care units, steps taken to enhance PEG practices can not only improve the quality of patient care but also ensure a more patient-centered approach in palliative care. In this regard, it is crucial for hospital palliative care units to develop necessary training, equipment, and procedures to make PEG applications more effective and accessible for patients. Consequently, this can lead to an enhancement in the quality of patient care and facilitate a more efficacious implementation of a patient-centered approach in palliative care.

Studies have shown that patients evaluated by an interdisciplinary team before PEG insertion experience accelerated treatment processes, reduced hospital stays, and alleviated concerns of patients and caregivers [15]. The home care and feeding of patients with a PEG in-situ consider the degree of malnutrition, the patient’s tolerance to nutritional support provided during the hospital stay, the desire to continue nutritional support at home, and the physical and social support of the caregivers. In Germany, out of 140,000 patients receiving PEG annually, 100,000 continue enteral nutrition support at home. Similarly, in the UK, about 265,000 patients with a PEG in-situ and in the USA, over 200,000 PEG patients annually continue home enteral nutrition support [16–17]. The aim in treating patients with a PEG in-situ at home is to improve their quality of life [15]. In the study, all patients with a PEG in-situ were transferred to home care units with support from an interdisciplinary team (dietitian, psychologist, social worker, physiotherapist, and home care technician) and a caregiver training program. We believe this training program played a significant role in reducing the anxiety and depression levels of caregivers and in the observed change between the initial HADS score at hospital admission and the score at discharge.

With the continuation of enteral nutrition at home, there is a decrease in costs, and the patient’s participation in family processes increases. Caregivers play a crucial role in the home care process of patients with a PEG in-situ. They encounter social, physical, psychological, and financial challenges while providing care. The costs associated with treatment and transportation create budgetary strains for both patients and caregivers. Many caregivers’ work lives are negatively impacted, with changes in working hours or even leaving their jobs. The social life of those involved in patient care is limited, leading to familial conflicts and the burden of care often falling on a single family member [18]. Additionally, this process can lead to restlessness, insomnia, and social isolation among caregivers [19]. Consistent with the literature, this study found that caregivers of patients in palliative care with feeding difficulties had high anxiety and depression risks from admission. This level decreased as their patients’ nutritional problems were resolved and palliative support was accessed, among caregivers of patients choosing PEG over other enteral methods. This suggests that when considering long-term feeding methods, PEG more significantly reduces the anxieties of caregivers of patients fed through other enteral methods, thereby lessening their care burden.

A study in Turkey found that about eight out of ten caregivers of home care patients are women, with an average age group of 45–65 [20]. The study observed that as caregivers age, their care burden increases, particularly leading to more psychological problems [21]. Another study on caregivers of hospitalized cancer patients found that male caregivers experienced less stress compared to female caregivers, and stress levels increased with age for both genders [22]. In the study, we found that the hospital anxiety levels of caregivers, irrespective of gender, were high, which decreased during their stay in the palliative service. However, as caregivers aged, this reduction rate decreased, and anxiety and depression risks remained higher. Therefore, we believe that particularly for older caregivers, more extensive psychosocial support from the interdisciplinary team and more effective clinical support services are needed.

## Conclusions

If a patient is to be fed enterally for an extended period, PEG should be the preferred method unless contraindicated, considering its advantages in effective and easy feeding for caregivers. Effective care training at PEG centers, psychosocial support from an interdisciplinary team, and supervision by home care technicians in PEG feeding and medication administration will reduce caregivers’ anxieties, help alleviate their psychological problems, and decrease their care burden.

## Data Availability

No datasets were generated or analysed during the current study.

## Abbreviations

**PEG**: Percutaneous Endoscopic Gastrostomy
**HADS**: Hospital Anxiety and Depression Scale
**HETF**: Home Enteral Tube Feeding
**ETF**: Enteral Tube Feeding
**IHAS**: Initial Hospital Anxiety and Depression Scale Score
**DHAS**: Discharge Hospital Anxiety and Depression Scale Score

## References

[1] Arai H, Ouchi Y, Toba K, Endo T, Shimokado K, Tsubota K, Matsuo S, Mori H, Yumura W, Yokode M, Rakugi H, Ohshima S (2015). Japan as the front-runner of super-aged societies: perspectives from medicine and medical care in Japan. Geriatr Gerontol Int.

[2] Ojo O (2015). The challenges of Home Enteral Tube Feeding: A Global Perspective. Nutrients.

[3] Özden D, Karagözoğlu Ş, Güler N, Bülbüloğlu S (2016). Problems related to Nutrition experienced by patients with Home Enteral Tube and the Care Burden of their relatives. DEUHFED.

[4] Harrington M, Lord L, Holcombe B (2005). Enteral nutrition implementation and management. The ASPEN Nutrition support practice manual.

[5] Shuremu M, Belachew T, Hassen K (2023). Nutritional status and its associated factors among elderly people in Ilu Aba Bor Zone, Southwest Ethiopia: a community-based cross-sectional study. BMJ Open.

[6] Arıkan Z, Erkal H, Özyurt Y, Yıldırım M. Total Enteral Nutrition. In: Kartal EA, editor. Kartal EA Med J. 2000;11(3):950–953.

[7] Adeyinka A, Rouster AS, Valentine M, Enteric Feedings. [Updated 2022 Dec 26]. In: StatPearls [Internet]. Treasure Island (FL): StatPearls Publishing; 2023 Jan-. Available from: https://www.ncbi.nlm.nih.gov/books/NBK532876/. 30422471

[8] Šare S, Ljubičić M, Gusar I, Čanović S, Konjevoda S (2021). Self-Esteem, anxiety, and Depression in older people in nursing homes. Healthcare.

[9] Özceylan G, Kolcu G. Biopsychosocial Status Assessment Tool for Patients and Relatives in Palliative Service. Avaliable from: https://www.researchgate.net/publication/360427732_Ozceylan_Palyatif_Servis_Hasta_ve_Hasta_Yakini_Biyopsikososyal_Durum_Degerlendirme_Araci.

[10] Gauderer MWL, Ponsky JL, Izant RJ (1980). Gastrostomy without laparotomy: a percutaneous endoscopic technique. J Paediatr Surg.

[11] Gomes CA Jr, Andriola RB, Bennet C, et al. Percutaneus endoscopic gastrostomy versus nasogastric tube feeding for adults with swallowing disturbances. Cochrane Database Syst Rev. 2015;5CD008096. 10.1002/14651858.CD008096.pub.4. 10.1002/14651858.CD008096.pub4 PMC6464742 25997528

[12] Stroud M, Duncan H, Nightingale J (2003). British Society of Gastroenterology. Guidelines for enteral feeding in adult hospital patients. Gut.

[13] Karabekiroğlu A, Demir EY, Aker S, Kocamanoğlu B, Karabulut GS (2018). Predictors of depression and anxiety among caregivers of hospitalised advanced cancer patients. Singap Med J.

[14] Topçuoğlu MA, Özdemir AÖ, Aykaç Ö (2022). Gastrostomy in hospitalized patients with Acute Stroke: NöroTek Turkey Point Prevalence Study Subgroup Analysis. Turk J Neurol.

[15] Pennington C. To PEG or not to PEG. Clin Med (Lond). 2002 May-Jun;2(3):250-5. 10.7861/clinmedicine.2-3-250. PMID: 12108477; PMCID: PMC4954042. 10.7861/clinmedicine.2-3-250 PMC4954042 12108477

[16] Wirth R, Bauer JM, Willschrei H, Volkert D, Sieber CC (2010). Prevalence of percutaneous endoscopic gastrostomy in nursing home residents–a nationwide survey in Germany. Gerontology.

[17] Kurien M, Westaby D, Romaya C, Sanders DS (2011). National survey evaluating service provision for percutaneous endoscopic gastrostomy within the UK. Scand J Gastroenterol.

[18] Rosa E, Lussignoli G, Sabbatini F, Chiappa A, Di Cesare S, Lamanna L, Zanetti O (2010). Needs of caregivers of patients with dementia. Arch Gerontol Geriatr.

[19] Papatya K, Sevinç KT, Çiltaş NY, Doğan M. IENSC. Care Burden and Social Support Levels of Caregivers in a Palliative Care Clinic. In: Proceedings of the International Health Sciences Conference (2018); 2018 Nov 14–17; Turkey.

[20] Taşdelen P, Ateş M (2012). The needs of Home Care patients and the burdens of their caregivers. J Educ Care Nurs.

[21] Selçuk KT, Avcı D (2016). Care Burden and influencing factors in caregivers of Elderly with Chronic Illness. Bandırma Onyedi Eylül. Univ J Health Sci.

[22] Kim Y, Baker F, Spillers RL (2007). Quality of life of cancer caregivers: effects of gender, relationship, and appraisal. J Pain Symptom Manage.

